# Esthetic Management in Geminated and Supernumerary Maxillary Incisor Teeth With a Conservative Interdisciplinary Approach

**DOI:** 10.1155/crid/9091745

**Published:** 2025-07-26

**Authors:** Mandana Karimi, Elham Ahmadi, Sepideh Arab, Pooya Raeesi

**Affiliations:** ^1^Department of Restorative Dentistry, Faculty of Dentistry, Alborz University of Medical Sciences, Alborz, Iran; ^2^Department of Restorative Dentistry, Faculty of Dentistry, Tehran University of Medical Sciences, Tehran, Iran; ^3^Department of Orthodontics, Faculty of Dentistry, Tehran University of Medical Sciences, Tehran, Iran; ^4^Department of Endodontics, Faculty of Dentistry, Alborz University of Medical Sciences, Alborz, Iran

**Keywords:** dental veneers, orthodontics, supernumerary tooth, tooth gemination

## Abstract

This clinical report describes an 18-year-old male patient that had geminated and supernumerary maxillary incisor teeth causing anterior dental crowding and esthetic problems. In this case, a conservative interdisciplinary approach that included digital analysis, supernumerary tooth extraction, orthodontic space management, and ceramic veneers was done. With these conservative multidisciplinary treatments and digital analysis, the vitality of the geminated tooth was maintained, and the esthetic, function, and patient self-esteem were improved significantly.

## 1. Introduction

Dental anomalies include abnormal variations in the number, morphology, size, structure formation, and eruption times of teeth that can occur in primary and permanent dentitions [1]. The etiology arises from different interacting factors ranging from genetic defects to environmental insults [2]. These anomalies can lead to esthetic, occlusal, and functional problems, and, as a result, early diagnosis and intervention in these cases are important [3]. The abnormalities in tooth size, form, and number include fusion, gemination, macrodontia, microdontia, and missing and supernumerary teeth [4].

Gemination or twinning occurs when a tooth germ divides partially or completely into two teeth. This results from an enlarged crown (megadont) that is usually characterized by a longitudinal crown fissure/notch [5]. In the most radiographic evaluation, a wide mesiodistal root with a wide pulp chamber is seen, which may have two root canals [5]. The etiology of gemination is uncertain, but it seems that complex genetic and environmental factors (such as trauma) are involved in its development [2]. The incidence of gemination is generally 0.1%–0.47% and is mostly observed in the anterior maxillary region and unilaterally [6]. Gemination is different from fusion, which refers to the merging of two teeth buds leading to a large crown with two distinct roots and a reduction in the number of teeth [6, 7].

Supernumerary teeth are another dental anomaly. These extra teeth can have similar or different normal tooth morphology that is classified into odontoma, conical type, tuberculate type, and supplemental teeth [8, 9]. The supplemental tooth has the same morphology as the normal tooth [8]. The anterior of the maxilla is the most common site that a supernumerary tooth is seen, and its prevalence is between 0.2% and 5.3% depending on the region of occurrence [9].

In the anterior region, both of these anomalies can lead to crowding, malocclusion, dental caries, periodontal esthetic, and functional problems [5, 10, 11]. The management of geminated and supernumerary teeth remains a major challenge in dentistry, and a multidisciplinary approach is advocated to balance patient needs with treatment possibilities and limitations [12, 13]. The comprehensive treatments usually involve endodontic, restorative, surgical, periodontal, and orthodontic management [12–14]. The best management is a conservative treatment that preserves the vitality of the pulp and provides the patient's functional and esthetic needs [12–14]. This clinical report describes conservative and esthetic interdisciplinary treatment in an adolescent patient with geminated and supernumerary teeth.

## 2. Clinical Report

**Figure 1:**
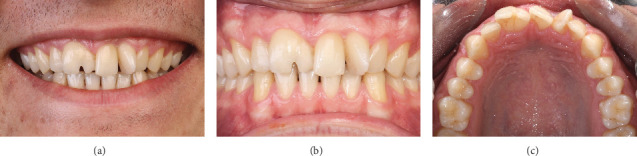
Clinical images of maxillary incisors demonstrate geminated right central and supernumerary tooth next to left central. (a) Smile view. (b) Retracted labial view. (c) Retracted occlusal view.

**Figure 2:**
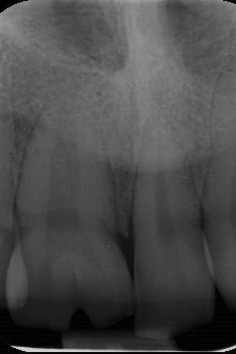
Radiographic image of the geminated right maxillary central showed a labial notch, leading to a partially bifid crown.

An 18-year-old male was referred to the Department of Restorative Dentistry at Tehran University of Medical Sciences with chief complaints related to the esthetic appearance of his maxillary anterior teeth. His medical and dental histories were noncontributory. Clinical and radiographic examinations revealed a geminated right maxillary central incisor exhibiting a single root and an enlarged pulp chamber, a narrow left central incisor, and an additional tooth located mesial to the left central incisor. The diagnosis of the supernumerary tooth was established through a combination of clinical and radiographic criteria. Although the tooth displayed close morphological similarity to the adjacent incisors, the increased anterior tooth count, its aberrant position and rotation, as well as subtle differences in crown form and root angulation—confirmed via periapical and panoramic radiographs—supported its identification as a supernumerary tooth. The geminated right incisor additionally exhibited a labial notch, resulting in a partially bifid crown (Figures 1 and 2).

Based on clinical and radiographic findings, extraction of the supernumerary tooth followed by orthodontic alignment and restorative correction was proposed as a conservative treatment plan, which was accepted by the patient. The decision to extract the supernumerary tooth was primarily based on its unfavorable position within the dental arch, including a 90° rotation and lack of proper alignment. In contrast, the adjacent central incisor demonstrated more favorable axial inclination, root development, and periodontal support. These factors, in addition to the esthetic benefit of preserving the better-positioned tooth, guided the treatment decision to retain the natural incisor and remove the supernumerary one.

**Figure 3:**
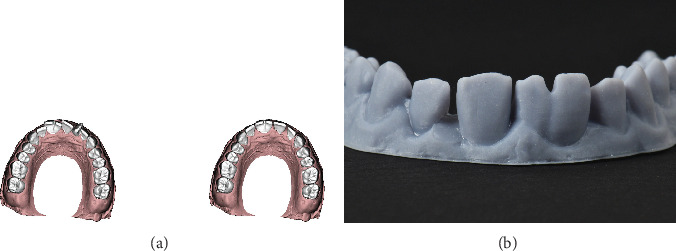
Digital analysis of maxillary arch. (a) Initial position of the teeth in the left side and final position of teeth after supernumerary tooth extraction and orthodontic space management according to Bolton's analysis in the right side. (b) The 3D-printed guided cast for orthodontic treatment was fabricated according to digital analysis.

Initial alginate impressions (Chromogel, Marlic) were made for diagnostic casts. Then, the diagnostic casts were scanned by a laboratory scanner (DOF, FREEDOM HD). According to the size of the patient's teeth, Bolton's analysis and the patient's midline, the ideal position of the teeth after orthodontic treatment was suggested by digital device (DOF, FREEDOM HD). This tooth position was 3D printed and delivered to the orthodontist for treatment guidance (Figure 3).

Orthodontic treatment was meticulously planned to preserve the labial alveolar bone and maintain optimal soft tissue contours. The bracket on the supernumerary tooth was positioned 1.5 mm gingivally relative to adjacent teeth to mitigate alveolar bone resorption and support gingival architecture. Notwithstanding these preventive measures, a certain degree of gingival asymmetry—primarily attributed to the intrinsic morphology of the left central incisor—was anticipated. This limitation was thoroughly communicated to the patient during the treatment planning phase, and the potential necessity for adjunctive periodontal interventions was discussed to ensure comprehensive esthetic management.

Since the treatment was adjunctive rather than comprehensive, and the amount of space to be closed was minimal, a round SS wire was selected to reduce friction and facilitate controlled tooth movement. Additionally, as the movement of the maxillary incisors was primarily mesiodistal, without significant retraction or torque demands, the use of a rectangular archwire was not necessary at this stage. Alignment and leveling were performed via 0.014, 0.016, and 0.018 NiTi wires and space closure was performed on 0.018-SS archwire using elastomeric chains.

**Figure 4:**
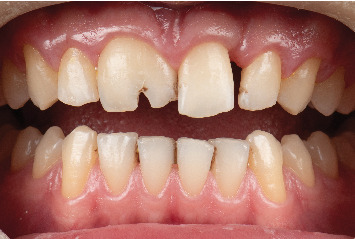
The maxillary teeth position after orthodontic treatment.

After 9 months, there was approximately 0 mm space on the mesial side and 2 mm space on the distal side of the maxillary left central incisor, and 1 mm on the distal side of the maxillary left lateral incisor, consistent with the 3D-printed cast (Figure 4).

**Figure 5:**
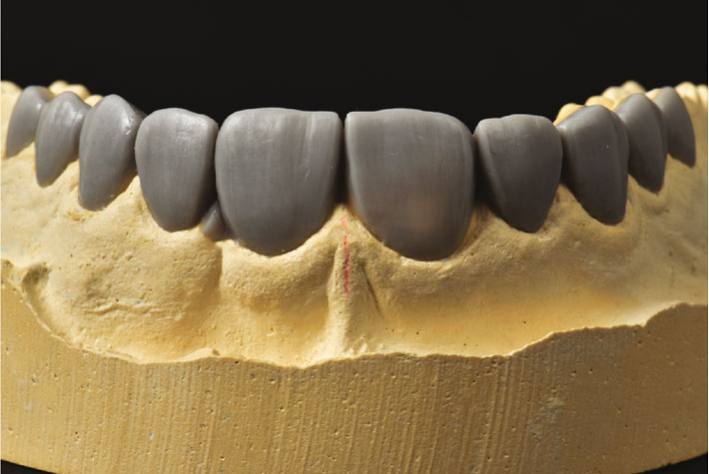
The diagnostic wax-up was done on the maxillary cast after orthodontic treatment.

After completion of orthodontic space management, the patient was referred for esthetic restorative treatment. Maxillary alginate impressions (Chromogel, Marlic) were obtained, and conventional wax-ups were performed on the cast (Figure 5). A silicone putty index (Putty, Panasil, Kettenbach, USA) was fabricated from the diagnostic wax-up and filled with a bis-acryl provisional material (TECO Provi KB, P.L. Superior Dental Materials); it was placed on the unprepared teeth to guide controlled and conservative tooth reduction.

The extent of restorative intervention was determined based on a combination of esthetic, functional, and patient-related factors. The patient expressed concern about potential shade mismatch and contour inconsistencies if only selective veneers were placed. Additionally, all maxillary incisors and first premolars (Teeth 14–24) were fully visible in the smile line, making comprehensive coverage necessary for achieving harmonious shade, shape, and gingival contour. From a functional standpoint, veneering this group of teeth allowed for proper canine guidance and reduction of lateral interferences, contributing to the long-term stability of the occlusion following space closure [15]. The rationale and implications of this approach were thoroughly discussed with the patient, and informed consent was obtained, emphasizing the minimally invasive nature of the preparation.

**Figure 6:**
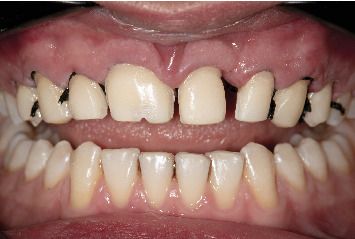
The maxillary teeth preparation for laminate veneers by using aesthetic pre-evaluation temporary (ATP) technique.

**Figure 7:**
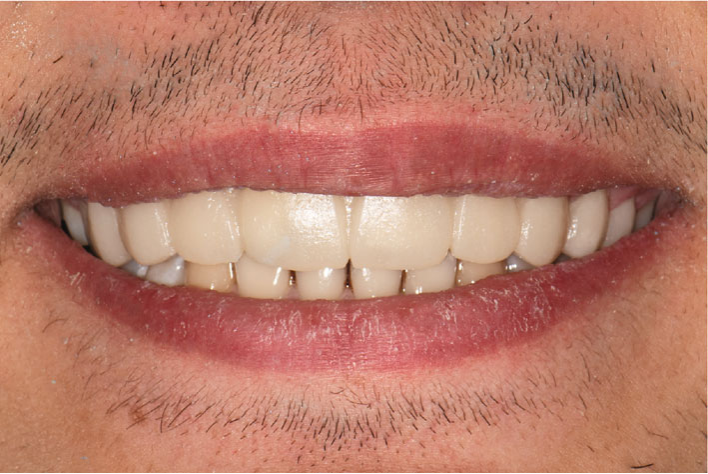
Bis-acryl temporary restoration on prepared teeth.

Tooth preparation was carried out using the aesthetic pre-evaluative temporary (APT) technique, with approximately 0.5 mm reduction on the facial/axial surfaces and 2 mm on the incisal edges, using a round-end tapered diamond bur (Jota Diamond Burs, Swiss) (Figure 6) [16, 17]. Gingival retraction cords (EasyCord 000; Müller-Omicron GmbH & Co. KG) were placed, and final impressions were made using a two-step putty-wash technique with polyvinyl siloxane material (Initial Light Contact and Putty; Panasil, Kettenbach, USA). A mandibular alginate impression (Chromogel, Marlic) and a bite registration (Futar D Slow; Panasil, Kettenbach) were also obtained. Provisional restorations were fabricated and placed immediately (Figure 7), with special attention to preserving interdental papillae—particularly between the left central and lateral incisors—to maintain gingival esthetics and prevent black triangle formation.

The layered lithium disilicate ceramic (IPS e.max Press; Ivoclar Vivadent AG) veneers were fabricated in a dental laboratory with A2 shade and medium translucency in the incisal edge. The ceramic veneers were bonded to the etched and isolated maxillary incisors and first premolars with a light cure resin luting cement (Translucent shade, Choice 2, Bisco) under isolation. Finally, orthodontic retention was performed by bonding a twist 0.0175 fixed retainer to the palatal side of the incisors after cementation of the laminates.

**Figure 8:**
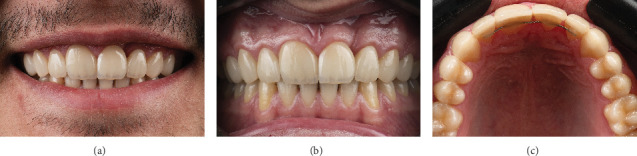
Clinical images of eight laminate veneers of maxillary teeth after the 1-year follow-up. (a) Smile view. (b) Retracted labial view. (c) Retracted occlusal view.

At the 1-year follow-up, clinical evaluation revealed healthy, coral-pink gingival tissue surrounding the maxillary incisors, with no evidence of inflammation, tooth sensitivity, or complications related to the laminate veneer restorations, such as caries, debonding, or chipping (Figure 8). Although complete gingival symmetry between the central incisors was not entirely attainable, the patient expressed satisfaction with both esthetic and functional outcomes and declined further periodontal procedures. Overall, the multidisciplinary approach effectively restored esthetics and function while maintaining periodontal health.

## 3. Discussion

The esthetic management of geminated and supernumerary maxillary incisors presents unique challenges due to the complexity of these dental anomalies, particularly in the anterior region where esthetics is crucial. Gemination, the incomplete splitting of a tooth bud, and supernumerary teeth, or additional teeth beyond the normal complement, can lead to issues such as irregular tooth morphology, spacing problems, and alignment disturbances [8]. These irregularities can significantly impact both the appearance and function of the dentition, often necessitating specialized management that prioritizes a balance of esthetics, function, and preservation of tooth structure [9].

The treatment plan for most cases of supernumerary erupted teeth is tooth extraction. Although in certain cases where the extra tooth is accompanied by a missing tooth, there may be a desire to preserve it. Orthodontics treatments are usually prescribed after that to manage the crowding or space problem [8–10].

There are some available treatment modalities for adult patients with geminated central incisors [5, 18]. The first option, which is fairly aggressive, is extraction and later implant/prosthodontic treatment [18]. This type of treatment should be the last option, and it is rarely mentioned in the articles [5, 18, 19]. The second one is more complicated, usually consisting of endodontic, surgical, and later prosthodontic treatment without orthodontic management [18, 20]. This type of treatment without orthodontic management is less conservative and leads to more tooth preparation and the possibility of endodontic treatment. The last and best option is a comprehensive treatment of orthodontic space management combined with prosthodontic treatment and, if necessary, surgical and endodontic treatments. Although this type of treatment is more time-consuming and expensive, it is the most conservative treatment option [18, 21].

In this case, a conservative interdisciplinary approach proved effective in addressing these challenges. The collaborative effort among orthodontics and restorative dentistry by using digital workflow allowed for a comprehensive and minimally invasive treatment strategy. This approach emphasized preserving natural tooth structure, avoiding overly aggressive interventions, and achieving harmonious esthetics, which aligns with contemporary principles of minimally invasive dentistry [22].

The integration of digital space analysis and 3D printing into esthetic planning in dentistry has revolutionized the field, allowing for precise treatment that enhances both function and appearance [23]. These technologies empower dental professionals to create accurate, customized treatment plans tailored to the specific esthetic goals of each patient. This shift is especially impactful in cosmetic and reconstructive dentistry, where digital tools can streamline the process, improve patient outcomes, and increase satisfaction [24].

Once the digital space analysis is complete, the digital data can be used to print guided casts or models of the patient's teeth. Traditional methods of creating models or guides for esthetic procedures could be labor-intensive and time-consuming [25]. With 3D printing, guided casts are produced quickly, accelerating the overall timeline for creating and fitting esthetic space management and restorations [26]. Combining digital space analysis and 3D printing creates a workflow that moves seamlessly from diagnosis and planning to implementation. Digital space analysis ensures that the treatment plan is based on precise data, while 3D printing ensures that the physical outcomes align closely with the plan. This predictability improves patient satisfaction, facilitates easier collaboration among dental specialists, and reduces unpredictable errors [27].

Occlusion plays a critical role in the long-term stability of diastema closure, particularly in cases involving significant anterior spacing. Inadequate occlusal guidance—especially the absence of proper canine guidance—can result in excessive functional loading on the anterior teeth during excursive movements, contributing to diastema relapse over time. Studies have emphasized that reestablishing ideal canine guidance facilitates posterior disclusion during lateral excursions, thereby protecting the midline contact from parafunctional forces and minimizing the risk of space reopening [15, 28]. Conversely, group function or unbalanced occlusal contacts in the anterior region have been associated with higher relapse rates, particularly in patients with prior orthodontic correction of midline spacing [29]. Thus, occlusal assessment and appropriate adjustment are crucial components of both the restorative and retention phases to maintain functional harmony and esthetic stability following diastema closure.

In patients with developmental dental anomalies—such as geminated or supernumerary teeth—gingival morphology is often compromised due to asymmetries in crown dimensions, abnormal emergence profiles, and alveolar bone irregularities [30]. These factors can impair the natural scalloping of the gingival margin and hinder the formation of ideal interdental papillae, particularly in the esthetic zone. The vertical fill of the interdental papilla is highly dependent on the distance between the bone crest and the contact point, with studies indicating that when this distance exceeds 5 mm, the likelihood of complete papillary fill and elimination of “black triangles” diminishes significantly [30, 31]. In this case, early preventive measures were taken to reduce the risk of black triangle formation, particularly in the esthetic zone. Careful shaping of the provisional restorations and deliberate modification of contact point position contributed to maintaining interdental papillae height and minimizing gingival embrasure visibility [31]. Despite anatomical limitations, these efforts were largely successful in preserving papillary fill, especially between the left central and lateral incisors.

Additionally, the gingival zenith symmetry of maxillary central incisors plays a significant role in smile esthetics, and even minor discrepancies may be perceived by patients. However, patient tolerance for such asymmetries can vary. Some studies have shown that discrepancies up to 1 mm in gingival margin height between central incisors may go unnoticed by laypersons, though dental professionals are more likely to detect even subtle differences [32, 33]. In our case, the gingival asymmetry—while clinically evident—was discussed preoperatively with the patient, who expressed satisfaction with the final result and declined further periodontal refinement. Given the morphological limitations and the patient's acceptance, no additional soft tissue interventions were pursued. This highlights the importance of individualized esthetic thresholds and shared decision-making in cases involving complex anatomical variation.

## Data Availability

The data that support the findings of this study are available from the corresponding author upon reasonable request.

## General Statement

*Clinical Significance*. The esthetic management of geminated supernumerary maxillary incisors poses unique challenges due to their complex morphology and potential impact on function and appearance. This article highlights the importance of a conservative interdisciplinary approach, involving precise analysis, minimally invasive procedures, and collaboration among dental specialties to achieve optimal esthetic and functional outcomes.
